# Immune remodeling of breast tumor-draining lymph nodes: antigen-presenting cell dysfunction, regulatory T-cell expansion, and premetastatic niche formation

**DOI:** 10.3389/fimmu.2026.1983258

**Published:** 2026-10-22

**Authors:** Liuhai Zeng, Haiyan Wu, Shihang Xue, Minzhi Chen, Jian Song, Wei Lin

**Affiliations:** Department of Breast and Thyroid Surgery, Xiangshan Hospital of Wenzhou Medical University, Ningbo, Zhejiang, China

**Keywords:** antigen presentation, breast cancer, premetastatic niche, regulatory T cells, sentinel lymph node, tumor-draining lymph node

## Abstract

Breast tumor-draining lymph nodes (TDLNs) support antitumor immune priming and frequently harbor the first regional metastases. Pathology-negative nodes can already show impaired dendritic-cell maturation and altered immune-cell positioning, whereas regulatory T-cell expansion becomes more prominent with increasing tumor burden. Lymphatic endothelium, fibroblastic reticular cells, sinus macrophages, and recruited monocytes further regulate antigen access, leukocyte trafficking, and tumor-cell retention. We review human and experimental evidence by nodal stage, breast cancer subtype, and treatment status. We propose a model in which priming competence and support for tumor colonization are related but partly separable dimensions of nodal remodeling. Altered cell positioning may weaken antigen presentation and immune surveillance. Stromal and regulatory feedback may then sustain suppression and favor outgrowth after tumor-cell entry. The order and causal coupling of these events remain unproven in human breast cancer. We distinguish candidate premetastatic alterations, detected before tumor cells at a stated detection limit, from functionally validated niche components that affect subsequent colonization. The model predicts that restoring antigen presentation and limiting stromal support can produce different effects on immune function, initial seeding, and later growth. We propose prospective sampling, antigen-specific functional assays, and timed perturbations to test these predictions and guide stage-specific biomarker development.

## 1 Introduction

Tumor-draining lymph nodes (TDLNs) receive lymph-borne antigens and support dendritic-cell (DC) activation and tumor-reactive T-cell expansion. Sentinel lymph nodes (SLNs) are the first nodes identified along the tumor’s drainage pathway; TDLNs encompass the wider draining network. These pathways also transport tumor cells. Axillary immune composition can predict disease-free survival beyond conventional nodal markers (1, 2).

Immune changes vary with nodal involvement. Among 70 clinically node-negative patients, metastasis-free SLNs had fewer human leukocyte antigen DR (HLA-DR)+ cells and fewer cells expressing the cluster of differentiation (CD) markers CD80, CD86, and CD40 than paired non-SLNs. Involved SLNs exhibited both type 1 and immunoregulatory signals (3). Subsequent studies localized defects mainly to node-resident DCs, with greater T-cell dysfunction at higher tumor burdens (4). Although TDLNs connect immunity and metastasis (5, 6), metastatic samples cannot establish which changes preceded colonization.

We examine how antigen-presenting cell (APC) abundance, positioning, and function contribute to impaired immunity. We then consider whether regulatory T cells (Tregs) initiate or amplify tolerance and whether stromal changes affect entry or outgrowth. Human studies establish clinical relevance, breast cancer perturbation models support causality, and other cancers provide mechanistic precedents (7, 8). **Figure 1** summarizes these relationships; Section 6.1 integrates them into a testable model.

**Figure 1:**
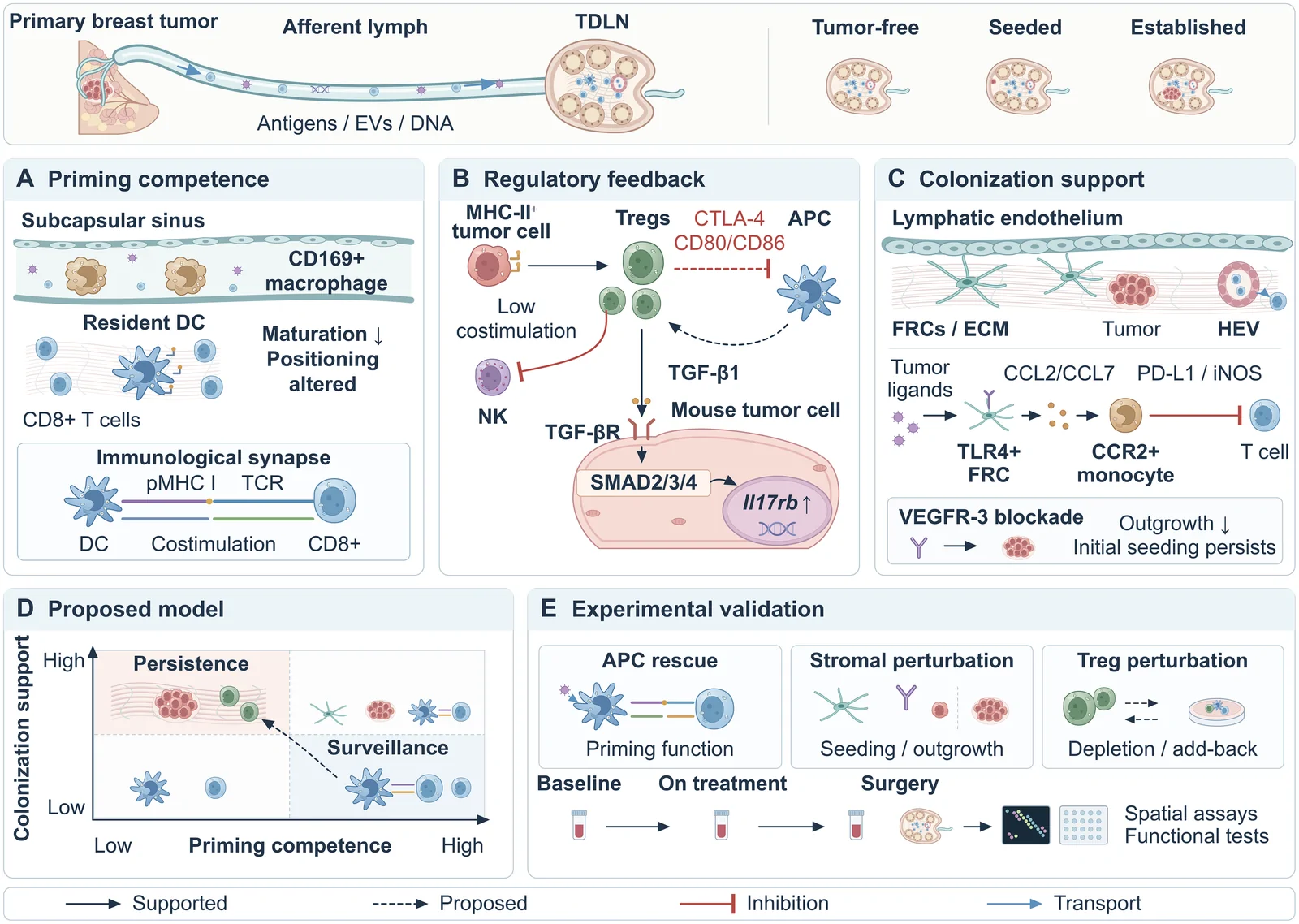
Immune and stromal remodeling in breast TDLNs. **(A)** APC dysfunction and altered positioning may weaken T-cell priming. **(B)** Treg-mediated immune suppression and tumor-cell signaling. **(C)** Stromal support for colonization; VEGFR-3 blockade limits outgrowth but preserves initial seeding in the MT-450 rat model. **(D)** Proposed model linking priming competence and colonization support. **(E)** Sampling and functional validation. Human associations remain distinct from causal model evidence. Black arrows, supported relationships; dashed arrows, hypotheses or proposed tests; red bars, inhibition; blue arrows, transport. EV, extracellular vesicle; iNOS, inducible nitric oxide synthase; pMHC I, peptide–MHC class I complex; TCR, T-cell receptor; TGF-βR, transforming growth factor β receptor.

## 2 Temporal and spatial remodeling of TDLNs in breast cancer

A tumor-free draining node does not, by itself, establish the presence of a premetastatic niche. We define candidate premetastatic alterations as reproducible tumor-induced changes preceding detectable tumor cells at a stated detection limit. Functional niche components must additionally affect subsequent colonization. Isolated tumor cells, micrometastases, macrometastases, and post-neoadjuvant nodes require separate analysis. PIK3CA-H1047R DNA is detected more frequently in metastasis-free SLNs than non-SLNs from mutation-bearing tumors (9). This finding indicates exposure to tumor material but does not establish the presence of viable cells or a functional niche.

Cell position can alter function independently of abundance. Afferent lymph enters the subcapsular sinus, where CD169+ macrophages capture particulate material. DCs occupy interfollicular and T-cell zones; fibroblastic reticular cell (FRC) conduits, lymphatic sinuses, follicles, and high endothelial venules (HEVs) regulate access. TDLNs contain smaller DC clusters, and tumor-positive nodes show fewer nearby T cells than healthy nodes (10). In estrogen receptor (ER)-positive/human epidermal growth factor receptor 2 (HER2)-negative disease, invasive lesions are associated with HEV expansion, discontinuous peripheral node addressin, and disorganized perivascular C-C motif chemokine ligand 21 (CCL21) (11).

Guo et al. compared untreated, metastasis-free TDLNs paired with seven immune-cold triple-negative breast cancers (TNBCs; stromal tumor-infiltrating lymphocytes below 10%) and eight immune-hot TNBCs (above 60%). The CD117-density validation analysis included 10 cases per group (12). Mature DC and T-cell abundance was similar, but cold-tumor nodes had higher type 2/type 1 helper T-cell (Th2/Th1) ratios, more DC–Th2 clusters, and more sinus interleukin-4 (IL-4)+ mast cells (12). The differences point to a role for spatial organization. Most other single-cell and spatial studies have examined established metastases (13, 14). Mass cytometry linked immune changes to deposit size (15); a single-patient study captured only 30 tumor cells (16). **Table 1** summarizes the findings by nodal context and identifies the limits of each study.

**Table 1:** Evidence for breast TDLN remodeling.

Sample and nodal context	Cell type	Key finding and mechanism	Evidence and subtype	Interpretation and limitation	Refs.
70 N0 patients; 56 metastasis-free SLNs paired with non-SLNs	DCs/APCs	Lower HLA-DR, CD80, CD86, CD40, CD83 and IFN-γ readouts; impaired maturation/costimulation	Flow cytometry, RT-PCR; multiple untreated subtypes	Paired-node evidence; cross-presentation not assessed	(3)
58 SLNs; 17 prophylactic controls. Separate ex vivo cohort: 29 SLNs.	Node-resident DCs; T cells	Resident DC activation impaired before involvement; T-cell dysfunction increases with burden	Flow cytometry and ex vivo assays; untreated breast cancer.	Peptide response in 4/9 hormone receptor-positive cases; processing rescue untested.	(4, 17)
15 paired untreated TNBCs; CD117 validation: 10 cases/group.	Mature DCs; Th1/Th2; mast cells	Higher Th2/Th1 ratio, DC–Th2 clusters and sinus IL-4+ mast cells in cold-tumor nodes; spatial association	NanoString, multiplex imaging; untreated TNBC	Cross-sectional; selected TIL extremes; validation size is assay-specific.	(12)
Tumor-free and metastatic SLNs	Tregs; IDO^+^ cells	FOXP3+ enrichment in metastatic SLNs; regulatory-cell enrichment	IHC; mixed subtypes	IDO lineage/causality not established	(23)
Presumed premetastatic TDLNs, week 11, spontaneous tumor model	T/B cells; FRCs	Treg-related programs and altered FRC metabolism; immune–stromal reprogramming	scRNA-seq; MMTV-PyMT, untreated	Timing-based; tumor-cell exclusion incomplete	(43)
TNBC TDLNs across tumor progression.	FRCs; CCR2^+^ monocytes	TLR4-dependent FRC CCL2/CCL7 recruits CCR2+ PD-L1-high suppressive monocytes	Mouse perturbation/spatial + human corroboration; TNBC	Strong in mice; human evidence correlational	(44)
Paired nodes from 6 patients; 474 validation sections from 58 patients.	CD169^+^ sinus macrophages	Fewer CD169+ macrophages in metastatic nodes; supported by spatial and histological analyses.	Transcriptomic, spatial, imaging; major subtypes	Within-patient association; spatial assays use selected sections and regions.	(20)
SLNs and nodal metastases across burdens	T cells; Tregs; LAMP3^+^ DCs; fibroblasts	Attenuated signaling in TIGIT+ effectors; activated Tregs increase with burden; lower inferred DC priming	Mass cytometry, single-cell/spatial; mixed subtypes	Correlational; small paired cohort	(18, 25)
Metastatic axillary nodes	CAF-S1; CAF-S4	Distinct CAF subsets promote migration/invasion (CXCL12/TGF-β, NOTCH)	Tissue, primary-culture assays; mixed subtypes	Explains established niches, not tumor-free FRC remodeling	(46)
Positive SLNs ± additional nodal involvement	Lymphatic endothelial cells	Lymphatic endothelial proliferation associates with additional non-SLN involvement.	IHC; early-stage, untreated	Post-colonization association; causality and premetastatic timing unresolved	(36)
Very early nodal metastases	30 tumor cells + adjacent niches	Six tumor-cell states; fatty-acid programs enriched in the dominant nodal cluster.	Single-case spatial transcriptomics	Single-case association; clonogenicity and pre-seeding state untested.	(16)
331 axillary nodes, 179 patients; 23 reactive controls	FRC network	FRC topology varies with tumor burden; outcome association is context-dependent	Digital pathology; post-neoadjuvant TNBC + others	Prognostic association, not mechanistic evidence	(41)

Evidence is classified by the sampled nodal state, assay, subtype, and treatment context. “Presumed premetastatic” denotes timing-based inference without sensitive exclusion of tumor cells in every node. Ex vivo rescue establishes reversibility under assay conditions, not clinical efficacy. The favorable outcome association for FRC topology applies to post-neoadjuvant, node-negative TNBC. IHC, immunohistochemistry; N0, clinically node-negative; RT-PCR, reverse-transcription polymerase chain reaction; scRNA-seq, single-cell RNA sequencing; TIL, tumor-infiltrating lymphocyte.

## 3 Dysfunction of antigen-presenting cells

### 3.1 Migration, maturation, and cross-presentation of dendritic cells

Assessing APC dysfunction requires more than cell counts. Priming depends on antigen processing, peptide presentation by major histocompatibility complex (MHC) molecules, costimulation, and contact with antigen-matched T cells. Type 1 conventional DCs (cDC1) specialize in cross-presentation, displaying acquired antigens on MHC I to CD8+ T cells; subset-specific human TDLN data remain limited (1). In 56 metastasis-free SLNs, HLA-DR+, CD80+, CD86+, and CD40+ populations were reduced relative to paired non-SLNs, with lower CD83 and interferon-γ (IFN-γ) transcripts in evaluable subgroups (3). These readouts reflect maturation but do not directly measure cross-presentation.

Flow cytometry of 58 SLNs (44 tumor-free, 14 involved), compared with 17 prophylactic-mastectomy axillary nodes, localized CD40, CD83, and CD86 downregulation mainly to resident conventional and plasmacytoid DCs (4). Effects on migratory CD1a+ DCs varied, with their frequency decreasing mainly in involved nodes. A separate ex vivo study of 29 SLNs combined B-class oligodeoxynucleotides containing unmethylated cytosine–phosphate–guanine motifs (CpG-B) with Janus kinase 2 (JAK2)/signal transducer and activator of transcription 3 (STAT3) inhibition. The combination enhanced mammaglobin-A peptide-specific T-cell responses in 4 of 9 evaluable hormone receptor-positive cases and was more effective than CpG-B alone (17). These findings indicate partial reversibility under culture conditions; peptide stimulation does not establish rescue of antigen processing or clinical efficacy.

A mature DC phenotype does not ensure productive contact with T cells. In tumor-positive non-SLNs, lower proportions of clustered DCs and mature DCs were associated with shorter disease-free survival (10). In eight paired tumors and nodal metastases, lysosome-associated membrane protein 3 (LAMP3)+ DCs showed weaker inferred priming programs in nodes (18). These transcriptomic scores do not directly measure antigen processing or antigen-specific contact. Transforming growth factor β1 (TGF-β1)-dependent DC apoptosis in lung cancer SLNs provides a cross-cancer precedent (19). Spatial measurements and antigen-specific activation assays are therefore needed alongside DC counts.

### 3.2 Sinus macrophages and B cells as accessory APC compartments

Sinus macrophages provide an antigen-capture barrier. A six-patient study compared paired non-metastatic and metastatic nodes and found a selective reduction in CD169+ macrophages in the spatially profiled samples. An additional 474 nodal sections from 58 patients supported this association (20). Because sampling occurred after metastasis, the direction of this relationship remains unresolved. In melanoma, these macrophages restrict vesicle access to cortical B cells (21); this mechanism remains untested in breast TDLNs.

In a mouse model of spontaneous breast cancer lymph node metastasis, TDLN B-cell immunoglobulin G (IgG) binds glycosylated tumor-cell heat shock 70 kDa protein 4 (HSPA4). This activates integrin β5 (ITGB5)/Src/nuclear factor κB (NF-κB) and enhances C-X-C motif chemokine receptor 4 (CXCR4)/stromal cell-derived factor 1α (SDF-1α) signaling and nodal dissemination (22). Antibody reactivity is associated with prognosis in humans, although this association does not establish the mechanism. Separately, forkhead box P3 (FOXP3)+ cells are enriched mainly in metastatic SLNs. Combined high FOXP3 and indoleamine 2,3-dioxygenase (IDO) staining identifies a subgroup, whereas IDO alone is not associated with nodal status and does not identify the responsible APC lineage (23).

### 3.3 Effects on productive T-cell priming

Profiling five paired tumors and SLNs yielded 65,285 cell transcriptomes and 15,831 T-cell receptor sequences (24). All SLN-expanded clonotypes occurred in tumors, whereas some tumor-expanded clonotypes were absent from sampled SLNs. This overlap cannot identify the site of initial priming. Across nodal burdens, T-cell immunoreceptor with immunoglobulin and immunoreceptor tyrosine-based inhibitory motif domains (TIGIT)+ effector T cells showed attenuated signaling, while activated Tregs increased (25). Lymphatic endothelial programmed death ligand 1 (PD-L1) deletes tumor-specific central-memory CD8+ cells in melanoma and colorectal models (26). This mechanism has yet to be validated in breast TDLNs.

## 4 Regulatory T-cell accumulation and tolerogenic feedback

### 4.1 Recruitment, retention, proliferation, and conversion

FOXP3+ enrichment occurs mainly in metastatic SLNs (23). In 50 patients with luminal A breast cancer, populations expressing programmed cell death protein 1 (PD-1), T-cell immunoglobulin and mucin domain-containing protein 3 (TIM-3), or FOXP3 varied by tissue compartment and nodal status (27). Cell density alone cannot distinguish recruitment, retention, proliferation, or conversion. Whether these observations extend to HER2-positive disease and TNBC also requires validation.

After nodal entry in mice, MHC-II-expressing cancer cells lacking adequate costimulation promote Treg proliferation and reduce effector CD4+ cells. Manipulating tumor-cell MHC II changes Treg expansion and nodal metastasis; MHC-II+ cancer cells also occur in human nodes (28). These findings concern remodeling during early metastasis and do not establish a mechanism operating in tumor-free nodes.

### 4.2 Reciprocal regulation of APCs and Tregs

Tregs can restrict costimulation through cytotoxic T-lymphocyte-associated protein 4 (CTLA-4)-mediated removal of CD80/CD86 (29). They can also produce interleukin-10 (IL-10) and transforming growth factor β (TGF-β), although the contribution of these cytokines depends on the tissue and response (30, 31). Tolerogenic APCs may reciprocally support Tregs (8). This loop remains incompletely validated across human nodal stages. In breast models, Treg depletion or TGF-β1 neutralization prevents acquisition of interleukin-17 receptor B (IL-17RB)-associated malignant programs by nodal tumor cells (31). Primary TNBC cancer-associated fibroblast subset 1 (CAF-S1) cells recruit, retain, and support CD25-high FOXP3-high cells (32). This offers a stromal precedent, although equivalent FRC behavior in tumor-free nodes has not been established.

CpG-B plus JAK2/STAT3 inhibition enhances SLN DC maturation and type 1 responses while limiting regulatory responses ex vivo (17). Under homeostatic conditions in mice, lymphatic endothelium presents self-antigens without sufficient costimulation and induces PD-1-dependent CD8+ deletion (33). The endothelial pathway provides a separate route to tolerance; these findings do not establish when APC–Treg feedback develops.

### 4.3 Functional and spatial heterogeneity

Treg phenotype and location may be more informative than density. Paired atlases resolve activated, follicular helper-like, and dysfunctional T-cell states, but inferred ligand–receptor interactions remain hypotheses (13, 18). In breast models, tumor-educated Tregs suppress nodal natural killer (NK) cells; depletion reduces nodal but not lung metastasis. Human SLNs also show increased Treg/NK ratios (34). Paracortical Tregs near DCs may affect priming, whereas Tregs beside metastatic nests may protect established tumor cells. Neither location establishes Treg origin.

## 5 Formation of premetastatic niches in breast cancer TDLNs

### 5.1 Tumor signals carried by lymph and lymphatic endothelial remodeling

Lymph carries tumor mediators, antigens, nucleic acids, and extracellular vesicles. PIK3CA-H1047R DNA in histologically negative SLNs indicates exposure before visible metastases (9). Vesicles can carry stable cargo, but evidence for specific vesicle–node pathways in human breast cancer remains limited (35). A functional niche requires a persistent response that affects subsequent tumor-cell survival or growth.

Among 65 SLN-positive patients, higher lymphatic endothelial proliferation within metastases was associated with additional non-SLN involvement (36). This association concerns changes after tumor-cell entry. In TNBC models, interleukin-6 (IL-6) activates endothelial STAT3/hypoxia-inducible factor 1α (HIF-1α) and induces C-C motif chemokine ligand 5 (CCL5) and vascular endothelial growth factor (VEGF) (37). In an MDA-MB-231-derived model, vascular endothelial growth factor C (VEGF-C) induces C-X-C motif chemokines, recruits C-X-C motif chemokine receptor 2 (CXCR2)-dependent myeloid cells, and promotes lymphatic invasion (38). In the MT-450 rat model, vascular endothelial growth factor receptor 3 (VEGFR-3) blockade reduces nodal outgrowth without preventing initial colonization (39). Entry and expansion therefore need to be measured separately.

### 5.2 Fibroblastic reticular cells, stroma, and myeloid programs

FRC networks and HEVs connect signaling to tissue organization. Invasive ER-positive/HER2-negative disease is associated with HEV dilation and disrupted addressin/CCL21 patterns (11). In 1,334 untreated nodes from 206 patients, follicular and cortical morphology correlated with location, tumor size, metastasis, and subtype (40). A 2026 study mapped 331 axillary nodes from 179 patients and 23 benign reactive controls using platelet-derived growth factor receptor β (PDGFR-β) and network metrics, including branching, alignment, and lacunarity (gap heterogeneity). The May 2026 correction clarifies that the association with favorable outcomes in node-negative TNBC applies to post-neoadjuvant nodes (41).

In melanoma TDLNs, tumor signals induce FRC proliferation and extracellular matrix (ECM) programs before overt metastasis, with reduced CCL21/interleukin-7 (IL-7) and abnormal immune positioning (42). In 11-week-old mice expressing polyomavirus middle T antigen under the mouse mammary tumor virus promoter (MMTV-PyMT), nodes sampled before usual detectable invasion show Treg-related programs and oxidative metabolic shifts in FRCs (43). This state remains presumed premetastatic because tumor cells were not sensitively excluded from every node. Signaling programs also differ between models.

TNBC models provide causal evidence for a breast-specific circuit. Tumor-associated Toll-like receptor 4 (TLR4) ligands induce FRC C-C motif chemokine ligands 2 and 7 (CCL2/CCL7) and recruit C-C motif chemokine receptor 2 (CCR2)+ PD-L1-high monocytes to vascular and T-cell regions (44). Monocytes suppress T cells through PD-L1 and inducible nitric oxide synthase. Local TLR4 inhibition plus PD-1 blockade reduces monocyte homing and lung metastasis in mice. Corresponding patterns in human TNBC nodes remain correlational. It is unclear whether this circuit also operates in other breast cancer subtypes.

### 5.3 From an immune-permissive state to metastatic colonization

Single-cell spatial analysis identified 30 keratin 19 (KRT19)+ cells across approximately 200 micrometers in one patient (16). Related tumor-cell clusters resolved six epithelial–mesenchymal states, with fatty-acid metabolism enriched in the dominant nodal cluster and immunosuppressive features nearby. These are transcriptomic associations, not functional tests of clonogenicity. This single case cannot determine whether the surrounding niche preceded seeding. Later atlases describe tumor heterogeneity and inferred escape pathways, including nectin cell adhesion molecule 2 (NECTIN2)–TIGIT (18, 45), which require perturbation-based validation.

Established lesions create physical and immune barriers. Nodal CAF-S1 and CAF-S4 promote migration or invasion through distinct pathways and are associated with distant recurrence (46). Across human cancers and spontaneous breast models, solid stress reduces functional HEVs and lymphocyte entry; stress relief restores access in mice (47). In mouse breast cancer models, VEGF-C+ macrophages redirect dissemination toward nodes rather than lungs (48). Nodal involvement predicts distant recurrence without establishing that every distant lesion originates in nodes (49).

## 6 Discussion and future directions

### 6.1 Priming competence and support for colonization

We propose organizing these findings around two partly separable dimensions. Priming competence describes antigen-specific T-cell activation supported by functional APCs and accessible cell contacts. Colonization support describes conditions favoring tumor-cell retention, survival, and outgrowth. Impaired resident DC activation and spatial immune polarization inform the priming dimension (4, 12). Stromal–monocyte suppression and separable VEGFR-3 effects on outgrowth inform the colonization dimension (39, 44). A node may lose priming competence before acquiring demonstrable colonization support.

Our central hypothesis is that coupling impaired priming to stromal support increases metastatic persistence. Tregs may reinforce this coupling after seeding through APC inhibition and NK-cell suppression (29, 34). Treg-derived TGF-β may further reinforce persistence through its effects on nodal tumor cells (31). The model predicts that APC rescue can restore T-cell activation without necessarily preventing entry, whereas stromal intervention may limit outgrowth despite persistent priming defects. These predictions require testing in humans, where subtype and treatment may alter either dimension.

The framework allows these mechanisms to interact without assuming that every node follows the same sequence. Priming competence could be quantified by antigen-specific activation and DC–T-cell contact, while colonization support requires tumor-retention or growth assays. If combined restoration of priming and disruption of stromal support offers no benefit over either intervention despite target engagement, the proposed functional coupling would be weakened. Co-occurring markers alone cannot establish functional coupling.

### 6.2 Biomarkers linked to nodal state

Candidate biomarker combinations should reflect both mechanism and nodal context. Measures worth testing include DC maturation and contact, activated Treg–APC balance, CD169+ sinus integrity, FRC architecture, and stromal–myeloid neighborhoods. These combinations have yet to be validated. A multiplex-imaging study that included 205 paired tumors and nodal metastases found that p53 and GATA binding protein 3 (GATA3) provided prognostic information beyond clinical classifiers (50). Within-patient node comparisons reduce host variability (20); prospective validation must stratify deposit size, subtype, and treatment.

### 6.3 Intervention timing and preservation of immunity

CpG-B/STAT3 modulation has ex vivo support (17). TDLN-directed VEGF-C/VEGFR-3 and TLR4–CCR2 interventions remain preclinical (39, 44). Treg interventions also remain preclinical in this setting (34). TDLN-directed vaccination or local immunomodulation with checkpoint blockade remains investigational (51). After neoadjuvant chemotherapy, four patient pairs showed BTG2-low mast-cell enrichment in resistant metastatic nodes; accompanying experiments linked these cells to Treg induction, fibroblast activation, collagen deposition, and resistance (52). The small cohort does not establish that treatment caused the observed remodeling.

In breast cancer models, antigen processing persisted while DC presentation weakened with falling nodal interleukin-1β (IL-1β); local IL-1β or adjuvant delivery to distal antigen-positive nodes restored responses (53). A separate study profiled 99,671 lymphatic endothelial cells from paired metastatic/distal nodes of nine patients. Metastatic nodes had fewer PD-L1+ sinus-floor and medullary subsets and more CD200+ HEY1+ capillary-like cells. Ex vivo antibody-blocking assays on nodal sections linked matrix Gla protein (MGP) to tumor-cell adhesion (54). Because these studies concern different stages, interventions should be assessed for both immune priming and antimetastatic effects.

### 6.4 Experimental and clinical validation

Prospective cohorts should pair primary tumor, blood, and anatomically mapped nodes. Where clinically indicated sampling permits, a pretreatment nodal biopsy should be paired with the same mapped node collected at surgery. Blood should be collected at baseline, an early treatment cycle, and surgery. Untreated surgical cohorts should be analyzed separately. Surgical comparisons should include SLNs and additional nodes available from the indicated procedure, with drainage position and tissue allocation recorded. Prespecify hormone receptor-positive/HER2-negative, HER2-positive, and TNBC strata, and account for multiple nodes within each patient. Each node should undergo serial sectioning and epithelial immunostaining, with the sampled fraction and detection limit reported. Molecular testing can supplement this assessment, but tumor DNA alone cannot establish viable-cell involvement.

Pretreatment-to-surgery pairs can assess treatment-associated changes, provided biopsy effects are accounted for. They cannot establish an untreated premetastatic sequence. Once a node is excised, its subsequent colonization cannot be observed. Prospective node-negative human cohorts can test associations with recurrence, while staged animal sampling and longitudinal imaging can test temporal precedence directly. Cross-sectional comparisons across deposit sizes can provide complementary evidence (15, 41).

Functional studies should compare sorted APCs exposed to intact antigen or cognate peptide. Antigen-specific T-cell proliferation, cytokines, and killing should then be measured with MHC-blocking controls. Because peptide loading bypasses processing, measurements of costimulation and viability help distinguish processing defects from downstream failure. Spatial contact measurements should accompany these assays. In matched tissue cultures, Treg depletion/add-back and pathway inhibition can test reversibility. In subtype-matched models, timing effects should be compared by perturbing FRC-TLR4/CCR2, Tregs, or DC activation either before or after detectable seeding. Include target-engagement and rescue controls, compare single and combined interventions, and measure entry, outgrowth, and distant metastasis separately (39, 44).

For window-of-opportunity studies, prespecify safety and changes in antigen-specific function as early endpoints. Nodal pathological response, viable deposit size, and residual cancer burden can assess activity. Evaluation of event-free survival requires adequately powered follow-up. An independent cohort should test whether spatial biomarkers add predictive value beyond subtype, baseline burden, and treatment. These proposed endpoints should not be treated as validated surrogates for survival.

## 7 Conclusion

Priming competence and support for colonization provide a way to organize TDLN remodeling. We hypothesize that their coupling, reinforced by regulatory and stromal feedback, favors metastatic persistence. The sequence remains unproven in humans. Stage-defined prospective sampling and functional rescue should distinguish exposure markers, causal niche components, and responses to established disease. Interventions should demonstrate their effects on antigen-specific immunity, initial seeding, and subsequent outgrowth separately.
